# The Prevalence and Severity of Misophonia in a UK Undergraduate Medical Student Population and Validation of the Amsterdam Misophonia Scale

**DOI:** 10.1007/s11126-020-09825-3

**Published:** 2020-08-23

**Authors:** Jay Naylor, Charlotte Caimino, Polly Scutt, Derek J Hoare, David M Baguley

**Affiliations:** 1 Hearing Sciences, Division of Clinical Neurosciences, School of Medicine, University of Nottingham, Nottingham, UK; 2 Nottingham NIHR Biomedical Research Centre, School of Medicine, University of Nottingham, Nottingham, UK; 3 Nottingham Audiology Services, Nottingham University Hospitals NHS Trust, Nottingham, UK

**Keywords:** Misophonia, Undergraduate., Medical student., Amsterdam Misophonia Scale.

## Abstract

Misophonia is a condition of abnormal emotional responses to specific auditory stimuli. There is limited information available on the prevalence of this condition. This study aimed to estimate the prevalence of misophonia in an undergraduate medical student population at the University of Nottingham. A secondary aim of this study was to assess the psychometric validity of the Amsterdam Misophonia Scale (A-Miso-S) questionnaire tool in this population. The A-Miso-S was administered online to medical students at the University of Nottingham. To assess the validity of the A-Miso-S, a factor analysis was conducted. To determine prevalence and severity the results of the questionnaire were quantitatively analysed using SPSS. Actor analysis was conducted. Free text responses to one questionnaire item were analysed using a thematic approach. Responses were obtained from 336 individuals. Clinically significant misophonic symptoms appear to be common, effecting 49.1% of the sample population. This is statistically significantly higher prevalence than previous studies have found (*p* < 0.00001). Using the classification of the A-Miso-S, mild symptoms were seen in 37%, moderate in 12%, severe in 0.3% of participants. No extreme cases were seen. The A-Miso-S was found to be a uni-factorial tool, with good internal consistency. This study has provided new information on misophonia and validity of the A-Miso-S questionnaire in a sample population of UK undergraduate medical students. The results indicate that misophonia is a phenomenon that a significant proportion of medical students experience though only a small subset experience it severely.

## Introduction

Individuals with misophonia are reported to experience strong emotional responses, primarily of anger, in response to specific auditory stimuli such as chewing [1, 2]. Some individuals also report involuntary replication of the sounds themselves, and experience invasive mental images such as mouths [3]. Misophonia is a relatively newly observed and under researched condition, which was first described as a reaction of the limbic and autonomic systems of the brain over and above the norm in response to specific auditory stimuli [4]; this then causes up-regulated neural connections between the limbic system and the auditory system.

A variety of methods have been used to treat misophonia with mixed results. In one low quality study, CBT was found to be effective for eliciting short-medium term improvements in sufferer’s ability to function [5]. A multitude of non-invasive psychiatric therapies have been employed to treat Misophonia including mindfulness, counterconditioning, comorbid therapy, coping strategies, dialect therapy and pharmaceutical treatments [2]. However, there are still no clinical practice guidelines for management of this condition. If misophonia is a highly prevalent disorder, then clear diagnostic tools and clinical guidelines need to be finalized and implemented to ensure the best treatment for all individuals.

Research which focuses on the prevalence of misophonia is scarce, however data is beginning to emerge. Wu et al. [6] found the prevalence of misophonia was 19.9% in a sample of 483 US undergraduates using an online questionnaire study. The researchers also noted a common occurrence of comorbid anxiety disorders, obsessive compulsive disorders (OCD), and low mood. Prevalence did not differ by ethnicity, age or gender. Another study of 415 Chinese undergraduates [7] also placed the overall prevalence at 20%, though noted that severely interfering symptoms (severe and extreme on the Amsterdam Misophonia Scale) only made up 6% of the sample. Again, it was observed that associations existed between general sensory sensitivities, low mood, anxiety and obsessive compulsive-type disorders. It was found that generalised symptoms of misophonia were common and linked to several domains of psychopathology.

Misophonia often co-exists alongside similar conditions from which it is important to differentiate. Sufferers of misophonia may also experience tinnitus and/or hyperacusis [8] with misophonia and hyperacusis often grouped together as Decreased Sound Tolerance. Tinnitus sufferers experience the perception of sound in the absence of external stimuli [9, 10] and it is typically associated with hearing loss. Hyperacusis on the other hand is increased sensitivity to sound which results in pain or the sufferer feeling uncomfortable, and is related to the intensity [9, 10]. Misophonia is distinct from other conditions of decreased sound tolerance as it is a response to specific sounds with an abnormally intense emotional response [11]. Misophonia may be linked in some instances to brain injury where patients experience decreased sound tolerance following an injury [11]. Misophonia has also been linked to autonomous sensory meridian response (ASMR) where, in contrast to misophonia, people experience pleasure and wellbeing in response to specific sounds [12]. Misophonia has been observed as a symptom of OCD [13] where patients experience severe feelings of horror and disgust towards people or objects producing certain sounds [14]. The Amsterdam Misophonia Scale [15] is an instrument used to determine the presence and severity of misophonia, and the tool that the A-Miso-S is derived from was originally designed for assessing OCD severity. Misophonia and Tourette’s syndrome were recently linked [16], where tics are triggered in response to auditory stimuli with a misophonic response also being elicited. This may be as a result of common areas of the brain involved in both conditions [16].

Functional magnetic resonance imaging (fMRI) has shown that misophonic sounds lead to increased blood flow to the anterior insular cortex (AIC) of the brain, a key area in mediating the perception of signals and the emotional processing associated with them [17]. Moreover, trigger sounds elicit visible physiological responses including raised heart rate and changes in the electrical resistance of the skin. This response is caused by emotional stress when the main centre of the salience network, which the AIC is main component of, shows increased blood flow and activity [17].

Considering the estimated prevalence of misophonia and misophonia-like symptoms in the population, it remains a largely understudied condition [18]. Despite researchers and scientists highlighting misophonia as a distinct clinical condition, it remains absent from psychiatric classifications, and sufferers fail to obtain a diagnosis from less-informed practitioners [19]. This absence of knowledge and any clinical guidelines is of further detriment to sufferers who can be mismanaged and remain effectively untreated for misophonia [20]. Though assessment tools like the A-Miso-S and the Misophonia questionnaire exits, there is not enough research into which are most effective yet [6].

Research indicates that medical students are a distinct group from a generalised undergraduate population [21–25]. They exhibit higher rates of anxiety, depression and chronic stress, above the rates seen in other students [21]. Moreover, their levels of emotional intelligence are reportedly lower than seen in students of other disciplines [21]; this can be associated with lower levels of physical and mental wellbeing [21–25]. The distinctive features in this demographic indicate that misophonia prevalence here could differ from a more generalised sample.

The current study aimed to assess the prevalence and severity of misophonia in a population of UK undergraduate medical students. A secondary aim of this study was to assess the validity of the Amsterdam Misophonia Scale (A-Miso-S).

## Methods

Ethical approval was obtained from the University of Nottingham Faculty of Medicine and Health Sciences Ethical Review Board.

### Design

A cross-sectional study was used to examine the prevalence of misophonia. An optional, anonymous, closed online questionnaire was used to assess misophonia prevalence and severity.

### Participants

Participants were recruited from the School of Medicine at the University of Nottingham. The study was advertised in person (at lectures), by poster advertisement, and on social media sites. Advertisements provided participants with a link for anonymous participation in the online questionnaire where they were screened to ensure they met the inclusion criteria for the study. Criteria for inclusion were that participants spoke English, were aged over 18 years and were University of Nottingham medical students. Participants were advised not to take part in the study if they thought there was a possibility of experiencing a severe negative emotional response to the content of the questionnaire. The demographic characteristics of the University of Nottingham medical student population were that they were young (97% aged 18–24 years), Caucasian (66%), Asian (18%) or black (8%), and the majority were female (73%). There were no incentives for participants.

### Materials

The Amsterdam Misophonia Scale (A-miso-S) is a 7-item subjective, self-report measure which uses a 5-point Likert scale for participants to rate their response to each item [15]. The final item of the questionnaire requires a free text response from the respondent. The A-Miso-S is an adapted version of the Yale-Brown Obsessive-Compulsive Scale (YBOCS) [15, 26]. Scores are categorised into ranges of sub-clinical (0–4), mild [5–9], moderate [10–14], severe [15–19] and extreme [20–24]. Total scores of 4 or less are considered subclinical, whereas scores of 5 or more are considered clinically significant. The scale assesses how much time people are preoccupied with the sounds, how much interference they have in daily activities, how much distress is associated with the trigger sound, how much effort a sufferer makes to resist thinking of the sounds, how much control sufferers have over their thoughts, and their avoidance of the stimuli.

A calculated necessary sample size of 58 was found to be required for sufficient statistical power in the study using a cross-sectional study sample size formula and values of 95% confidence, 20% estimated prevalence (based on previous studies [6, 7]) and 5% precision [27]. The functionality of the survey tool as tested by the principal investigator prior to its release to participants.

### Procedure

Participants were provided with a participation information sheet with relevant details. They then proceeded to the questionnaire and consented to participate. Participants then completed each item and submitted the questionnaire. The collective results were exported to Excel for analysis. Data collection was done between the 22nd of October 2019 and the 4th of November 2019.

### Analytical Strategy

Statistical analyses were carried out using IBM SPSS Statistics 26 (Routeledge, Armonk NY).

Statistical significance was set at 5% for all statistical testing.

Prevalence of clinically significant misophonia was presented as a proportion with 95% confidence interval and was compared to a similar study (REF) using the z-test. For each question of the A-Miso-S, scores were summarised using the mean, SD, median, mode and frequencies of endorsement for each option. A-Miso-S scores in responders to the free text question were compared to score of non-responders using a Mann-Whitney-U test.

A thematic analysis guided by Braun and Clarke [28] was undertaken to analyse the final item of the survey which was an open-ended text-based response in order to deduce key themes that people with and without the condition experience to provide the study with a qualitative element.

A-Miso-S questionnaire data were subjected to a factor analysis which involved calculation of KMO values to determine if a factor Analysis was worthwhile, Chronbach’s alpha to measure agreement of questions and an initial exploratory factor analysis to produce a scree plot. Correlation matrices were produced by conducting a more detailed factor analysis with the data from the exploratory testing. Kendal’s Tau was conducted to assess the significance of correlations. Residual correlation testing was also conducted. There was no missing data to account for.

## Results

### Descriptive Statistics

Data was obtained from 336 participants who completed the questionnaire. The mean, standard deviation (SD), mode, median and frequency of endorsement of each answer option to the six scored questions of the Amsterdam Misophonia Scale are presented in Table 1.

**Table 1.** Scores per question with averages

					Frequency of endorsement of each score:				
Question	Mean	SD	Mode	Median	0	1	2	3	4
How Much of your Time Is Occupied by Misophonic Sounds	0.89	0.68	1	1	94	187	52	3	0
How Much Do these Misophonic Sounds Interfere with your Social, Work or Note Functioning?	0.59	0.70	0	0	175	129	27	5	0
How Much Distress Do the Misophonic Sounds Cause you?	1.10	0.83	1	1	176	79	57	20	4
How Much Effort Do you Make to Resist the (Thoughts about the) Misophonic Sounds?	0.80	1.00	0	0	78	169	71	15	3
How Much Control Do you Have over your Thoughts about the Misophonic Sounds?	1.14	1.03	1	1	106	114	73	36	4
Have you Been Avoiding Doing Anything, Going any Place, or Being with Anyone because of your Misophonia?	0.42	0.75	0	0	238	60	34	2	2

NB: Scores correspond to, 0 = None, 1 = Mild, 2 = Moderate, 3 = Severe, 4 = Extreme.

**Table 2:** Proportions in each category

Group:	n, (%)	95% Confidence Interval:
Subclinical (Total Score 0–4)	171 (51%)	0.45–0.56
Mild (Total Score 5–9)	124 (37%)	0.32–0.42
Moderate (Total Score 10–14)	40 (12%)	0.091–0.017
Severe (Total Score 15–19)	1 (0.3%)	0.00–0.016
Extreme (Total Score 20–24)	0 (0%)	0.00–0.011 (One-sided 97.5% confidence interval)

The proportions of individuals in each category are given in Table 2.

Overall, 49.1% (95% CI 0.44–0.55) of the 336 respondents had clinically significant misophonia (scores in the mild to extreme categories (total score: 5–24)) with the other 50.9% (CI 0.45–0.56) being sub-clinical (total score: 0–4). The proportion of individuals experiencing clinically significant misophonia in this study was compared to the proportion found in a study by Wu et al. [6] in 2014 (which noted clinically significant symptoms to be 19.9% (95% CI) in 483 participants) using a z-test, this study showed a statistically significant difference (Z score = −8.82, *p* < 0.001).

### Qualitative Analysis

**Fig. 1:**
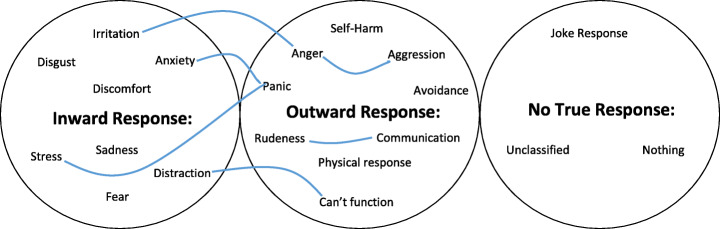
Mind map of themes showing links between codes

Regarding the responses to the free text question, it was determined that those who responded (*n* = 253) had statistically significantly higher scores than those who did not (*n* = 83) in misophonia severity (Mann-Whitney U test *p* = 0.024). Upon doing a thematic analysis of the resulting comments, 21 main codes were highlighted, including an unclassified category. These codes were then organised into three broader themes which became evident when looking at the essence of these comments. The codes and themes as well as some of the links between codes and themes in Fig. 1.

The thematic analysis indicated that respondents fell into two categories. Firstly, individuals who experienced an *inward response* to the stimuli felt an emotion represented by one of the codes in this theme such as irritation or distraction. Their response however, was not severe enough for them to take any outward action to stop it or remove themselves from the scenario, rather they just chose to tolerate it. On the other hand, some individuals whose responses were so severe that they were unable to tolerate the stimuli and so were forced to take action to stop or avoid the stimuli. Sometimes these *outward responses* such as aggression or being unable to function normally were what the respondents themselves were most fearful of experiencing. In addition to these we observed respondents who didn’t experience any misophonic symptoms as well as a few who made jokes or non-serious replies to the question. This highlights how misophonia is not recognised for the potentially disabling and crippling condition it is, if it were felt acceptable for medical students to joke about it.

### Factor Structure of the A-Miso-S

**Fig. 2–:**
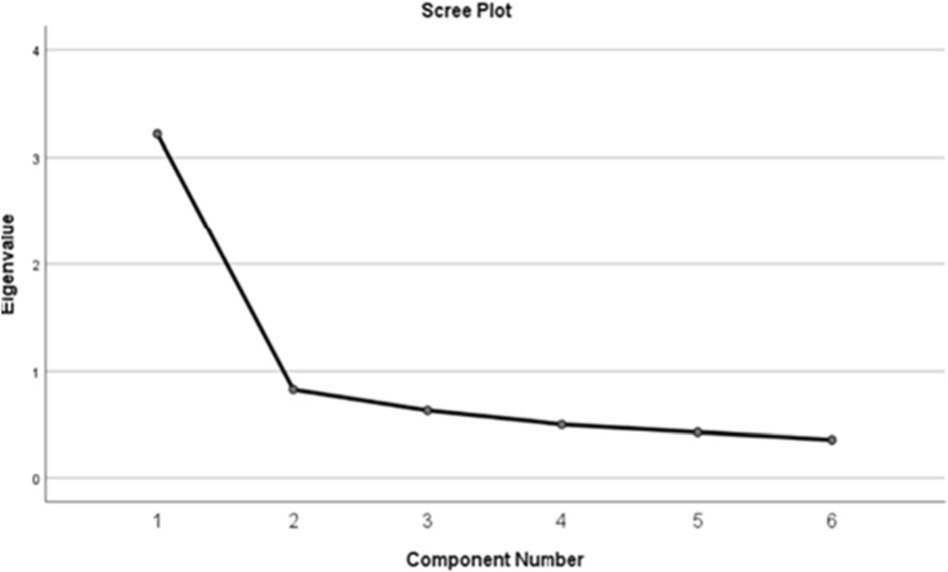
Scree plot illustrating the unifactorial nature of the A-Miso-S

Initial testing with the Kaiser-Meyer-Olkin measure of sampling adequacy gave a value of 0.847. A value of 0.814 was obtained for Chronbach’s alpha, this is a measure of agreement across all questions in the questionnaire, I.e. internal consistency. Initial exploratory factor analysis produced a scree plot (Fig. 2) which indicated only one factor was present. A single point on the plot had an Eigenvalue over 1 which was to the left of the index point.

In the light of the unifactorial structure, a correlation matrix was produced which describes how individual questions correlate with each other. The correlation matrix in Table 3 shows positive correlations exclusively (indicating a high score in one question increases the likelihood to give a high score in another question). Moreover, all the correlations were moderate, correlations over 0.5 would be strong, and these were all similarly correlated with values ranging from 0.209 to 0.593. Moreover, in conducting a Kendal’s Tau test it was shown that all of the correlations were statistically significant (*P* Value <0.001).

**Table 3:** . Correlation matrix

Question:	2	3	4	5	6	7
2	1.0	0.528	0.535	0.342	0.496	0.332
3	0.528	1.0	0.551	0.356	0.469	0.452
4	0.535	0.551	1.0	0.359	0.593	0.459
5	0.342	0.346	0.359	1.0	0.491	0.209
6	0.496	0.469	0.593	0.491	1.0	0.400
72	0.332	0.452	0.459	0.209	0.400	1.0

Tests for residual correlations were also conducted, these being the matrix of the difference between the correlations in the raw data and the correlations from the rotated factor space. The values that were obtained were between 0.1 and 0.001. Testing for total variance explained indicates how much of the variance was explained by one factor. The one factor that was found had an eigenvalue of 2.7 and contributed 45% of the variance in the sample with the other 55% being other variables such as random variation between participants and other sources (see Table 4).

**Table 4:** . Table of the variance explained by the factor

Factor	Total	% of Variance	Cumulative %	% of Variance
1	3.219	53.648	53.648	45.037
2	0.833	13.876	67.524	
3	0.638	10.629	78.153	
4	0.508	8.462	86.615	
5	0.437	7.277	93.892	
6	0.366	6.108	100.000	

## Discussion

This online self-report study investigated the prevalence of misophonia in a population of medical students using the A-Miso-S. The results highlighted that nearly half of the cohort experienced clinically significant symptoms.

The differences between the findings of the present study and that of Wu et al. [6] are substantial, with the present investigation finding a much higher prevalence of misophonia, though there are some important factors to consider as to why this may be. Wu and colleagues used the Misophonia Questionnaire (MQ) [6] as the A-Miso-S and other tools were not available at the time, and so the results are not directly comparable. Moreover, the studies were conducted in different countries (UK and the USA), and Wu et al. examined a generic population of undergraduates rather than a distinct population of medical students. This may partially account for higher levels of misophonic symptoms in medical students as this is consistent with the propensity to be more prone to conditions commonly co-occurring with misophonia.

Looking at the responses to each question, the majority of scores were low, any individuals scoring a 4 on a single question was very uncommon with most people scoring mildly in most questions and a smaller group scoring consistently moderately across all domains. The trend of the data may indicate that misophonia or misophonic symptoms are just a part of the normal human experience but that in some individuals these emotions and responses were heightened to a level that they are causing adverse emotional and behavioral effects.

The thematic analysis highlights the broad spectrum of different responses within misophonic individuals and provides insight into their coping mechanism which may be important for future research looking to find the most effective ways of mitigating misophonic impulses especially in individuals with particularly severe or disruptive responses.

There are some limitations to the present study. Selection bias may have affected the results as there is an approximate 40% increase in likelihood for people to cooperate with a study if they are interested in it or affected by it [29]. This may have artificially inflated the number of those with clinically significant misophonia that were detected. On the other hand, potential participants were encouraged not to partake if it might be distressing for them, so some of the more extreme cases of misophonia in our population may have been missed. Additionally, there was a non-response bias at play in the text box question. No data was collected in the respondents age, ethnicity, nor gender. The lack of the data on gender and age in particular means one is unable to assess exactly how representative the study population was to the overall medical student population. It also means one cannot draw any deductions of the role of age, gender, race or other factors on misophonia prevalence or severity. Additionally, there was no way to assess if an individual completed the questionnaire more than once, thus we cannot confirm all responses are from different individuals which could compromise the integrity of the results.

This study potentially indicates a seemingly un-met need in misophonic medical students for support. This also indicates that misophonia is a condition which effects many people mildly and a few people very severely. Furthermore, the A-Miso-S was found to be a robust measure of misophonia in medical students and accurately quantified a single construct. Overall, this condition appears to more common in this population than prior research might have suggested and so Medical Schools may need to improve their strategies surrounding it and this study should also encourage further research into diagnostic tools, treatment options and finding the best interventions for sufferers.
